# Dynamic changes in genome-wide histone H3 lysine 4 methylation patterns in response to dehydration stress in *Arabidopsis thaliana*

**DOI:** 10.1186/1471-2229-10-238

**Published:** 2010-11-05

**Authors:** Karin van Dijk, Yong Ding, Sridhar Malkaram, Jean-Jack M Riethoven, Rong Liu, Jingyi Yang, Peter Laczko, Han Chen, Yuannan Xia, Istvan Ladunga, Zoya Avramova, Michael Fromm

**Affiliations:** 1 Center for Biotechnology, 1901 Vine St., University of Nebraska, Lincoln, NE, 68588, USA; 2 School of Biological Sciences, University of Nebraska, Lincoln, NE, 68588, USA; 3 Department of Statistics, University of Nebraska, Lincoln, NE, 68588, USA; 4 Department of Agronomy & Horticulture, University of Nebraska, Lincoln, NE, 68583, USA; 5 Creighton University, Department of Biology, 2500 California Plaza Omaha, NE 68178, USA; 6 Department of Biomedical Engineering, University of California, Los Angeles, CA, 90095, USA; 7 Microsoft, One Microsoft Way, Redmond, WA 98052, USA

## Abstract

**Background:**

The molecular mechanisms of genome reprogramming during transcriptional responses to stress are associated with specific chromatin modifications. Available data, however, describe histone modifications only at individual plant genes induced by stress. We have no knowledge of chromatin modifications taking place at genes whose transcription has been down-regulated or on the genome-wide chromatin modification patterns that occur during the plant's response to dehydration stress.

**Results:**

Using chromatin immunoprecipitation and deep sequencing (ChIP-Seq) we established the whole-genome distribution patterns of histone H3 lysine 4 mono-, di-, and tri-methylation (H3K4me1, H3K4me2, and H3K4me3, respectively) in *Arabidopsis thaliana *during watered and dehydration stress conditions. In contrast to the relatively even distribution of H3 throughout the genome, the H3K4me1, H3K4me2, and H3K4me3 marks are predominantly located on genes. About 90% of annotated genes carry one or more of the H3K4 methylation marks. The H3K4me1 and H3K4me2 marks are more widely distributed (80% and 84%, respectively) than the H3K4me3 marks (62%), but the H3K4me2 and H3K4me1 levels changed only modestly during dehydration stress. By contrast, the H3K4me3 abundance changed robustly when transcripts levels from responding genes increased or decreased. In contrast to the prominent H3K4me3 peaks present at the 5'-ends of most transcribed genes, genes inducible by dehydration and ABA displayed atypically broader H3K4me3 distribution profiles that were present before and after the stress.

**Conclusions:**

A higher number (90%) of annotated Arabidopsis genes carry one or more types of H3K4me marks than previously reported. During the response to dehydration stress the changes in H3K4me1, H3K4me2, and H3K4me3 patterns show different dynamics and specific patterns at up-regulated, down-regulated, and unaffected genes. The different behavior of each methylation mark during the response process illustrates that they have distinct roles in the transcriptional response of implicated genes. The broad H3K4me3 distribution profiles on nucleosomes of stress-induced genes uncovered a specific chromatin pattern associated with many of the genes involved in the dehydration stress response.

## Background

Plants respond to external signals by activating signaling pathways that rapidly alter physiological reactions and transcription rates of responding genes. The molecular mechanisms regulating gene expression are coordinated at the genome level where the accessibility of DNA sequences is determined by the structure of chromatin. Chemical modifications of the histone amino-terminal tails are implicated in facilitating nucleosome remodeling and repositioning, and in the recruitment of specific transcription factors. Changes in DNA methylation and/or histone modifications are associated with altered gene expression under various stresses in plant and cell culture systems [1-5]. Changes in nucleosome occupancy and in the levels of histone H3 tri-methylation (lysine 4) or acetylation (lysine 9, 14 or 27) during dehydration stress occurred at four inducible *Arabidopsis *genes [6]. Dehydration stress induces biosynthesis of abscisic acid (ABA), which as a second messenger activates signaling cascades that trigger stomatal closure and altered gene expression via chromatin remodeling. In turn, ABA regulation is achieved through genetic and chromatin modification mechanisms [6,7]. Thus, chromatin structure and chromatin modifications are emerging as critical factors in plants' responses to environmental cues.

It is important to note, however, that currently available data describe histone modifications only at individual stress-induced plant genes. We also have limited knowledge of chromatin modifications taking place at genes whose transcription has been down-regulated. Moreover, there are no data on global modification trends occurring during the reprogramming of the entire genome in response to stress.

Here, we provide a genome-wide view and analysis of the histone H3 lysine 4 mono-, di-, and tri-methylation (H3K4me1, H3K4me2, H3K4me3, respectively) patterns in chromatin isolated from Arabidopsis rosette leaves before and after dehydration stress. Genome-wide transcript patterns in watered and dehydration stressed plants were compared with changes in the H3K4me1, H3K4me2, and H3K4me3 levels of nucleosomes associated with responding genes. Using chromatin immunoprecipitation (ChIP) with H3K4 methylation specific antibodies and genome-wide sequencing (ChIP-Seq), we revealed different dynamics and different magnitudes of the changes in H3K4me1, H3K4me2, and H3K4me3 profiles taking place upon dehydration stress. We demonstrate specific patterns of the H3K4me1, H3K4me2, and H3K4me3 distributions at up-regulated, down-regulated, and unaffected genes during the stress.

## Methods

### Plant materials and growth conditions

Col-0 plants were grown in 12 h light for 4 weeks in pots with soil covered with miracloth to prevent soil contamination of leaf tissues at harvest. Control plants continued to receive water while water was withheld from water-deficit treated plants until a relative water content (RWC) of 65% was reached in 4 to 6 days. Vegetative rosettes were harvested and frozen for RNA isolation or immediately fixed in formaldehyde for isolation of chromatin as described below.

### Isolation and immunoprecipitation of chromatin

Watered or water-deficit treated vegetative tissues were fixed in 1% formaldehyde for 10 min after vacuum infiltration of the tissues in phosphate buffered saline (PBS) with 0.007% silwet. Fixation was stopped by quenching with 125 mM glycine. Next, the tissue was rinsed twice in ice cold water, blotted dry and flash frozen in liquid nitrogen. Chromatin was isolated and resuspended in 300 μl nuclei lysis buffer as described by [8] and sonicated for four 15 sec pulses using a Branson sonifier 450 at an output between 10 - 15%. The sheared chromatin was used in chromatin immunoprecipitation assays as described [8] with some modifications. In brief, 100 μl of sheared chromatin was diluted in 900 μl ChIP dilution buffer and precleared, using 15 μl of protein A Dynabeads (Invitrogen, Carlsbad, CA). Protein A beads were collected using a magnet, and the precleared chromatin was incubated overnight at 4 °C with antibodies against either H3 (6 μL, Abcam ab1791, Cambridge, MA), mono-methylated H3K4 (10 μL, Abcam ab8895, Cambridge, MA), dimethylated H3K4 (6 μL, Upstate 07-212, Millipore, Billerica, MA), or trimethylated H3K4 (6 μL, Abcam ab8580, Cambridge, MA). Western blotting of the H3K4 methylation antibodies against mono-, di- or tri-methylated H3K4 or monomethylated H3K9 synthetic peptides was performed and confirmed their expected specificity (Additional file 1, Figure S1). A sample without antibody addition was also used as a control. Next the antibody/chromatin complex was precipitated by incubating the mix with 40 μl of protein A beads for 1 h followed by collection of the beads using a magnet. The beads were washed, chromatin eluted, and DNA de-crosslinked using procedures as described [8]. DNA was recovered by phenol/chloroform extraction and ethanol precipitation in the presence of Novagen pellet paint (EMD Chemicals, Gibbstown, NJ) and resuspended in 30 μL of TE buffer. Purified DNA was end modified, ligated to amplification primers, amplified, and sequenced according to the manufacturer's protocols (Illumina, San Diego, CA).

### RNA isolation and labeling

Total RNA was extracted from the frozen rosette tissues using grinding and TRIzol (Invitrogen, Carlsbad, CA) reagent. Three independent replicates of watered and dehydration-stressed tissues were used. Fifteen μg of total RNA from each sample was used to produce labeled cRNA for hybridization to Affymetrix Arabidopsis ATH microarrays using the manufacturer's protocols (Affymetrix, Santa Clara, CA).

### Microarray data analysis

Affymetrix^® ^GeneChip Operating Software (GCOS) generated the image and probe set signal values as CEL files, which were loaded into Rosetta Resolver^® ^(version 6.0, build 6.0.0.311; Rosetta Inpharmatics LLC, Seattle, WA). The water deficit and control condition replicate arrays were combined into their respective intensity profiles, and subsequently used to generate ratio experiments in Rosetta Resolver and analyzed using the software's background correction, normalization, and proprietary ratio error model [9]. The gene expression arrays have been submitted to the NCBI Gene Expression Omnibus under GSE11538 (part of SuperSeries record GSE11658).

### Computational Methods for the analysis of sequence data

The primary analysis of the Illumina Genome Analyzer output was performed by the manufacturer's ELAND program. ELAND mapped sequencing reads of 35 bases uniquely to the Arabidopsis genome with no more than 2 mismatches. As a quality control, we used BLAST [10] to re-map all reads to the Arabidopsis genome [11] with a word length of 9 bases and low-complexity filtering turned off. This analysis revealed a small number of further non-unique sequencing reads. From the remaining reads that uniquely map to the genome with no more than 2 mismatches, the coverage of each genomic position by the sequencing reads was computed. The DNA sequencing reads for total genomic DNA from *Arabidopsis thaliana *cultivar Col-0 were obtained and mapped to the *Arabidopsis thaliana *Col-0 genome, which is used as the control data set. To check for biases in Illumina sequencing, the distribution of sequencing coverage along the genome was calculated. Statistical analyses were performed in MATLAB (MathWorks, Nantucket, MA). Tests for the statistical significance in the difference in the coverage profiles of H3K4me3 along the normalized gene length between the sets of all expressed and ABA induced genes under both watered and soil water deficit conditions were performed by the non-parametric Wilcoxon rank sum test on the total (peak-normalized) coverage over the normalized gene length for individual genes.

Coverage peaks were identified as maximal scoring segments using a modified dynamic programming algorithm, analogous to pairwise local alignments [12]. Background noise was estimated as the mean coverage of the intergenic regions (ranging from 1.10 to 3.25 depending on the sequencing run) and was subtracted from the raw coverage values. We extended peaks from each genomic position that had an above background coverage so as to reach the maximum of the cumulative coverage, analogous to the cumulative score of a BLAST High-Scoring Segment Pair. During peak extension, we allowed for local drops in the peaks provided that they were compensated by subsequent above-background regions.

Position-wise coverage data for the three H3K4 methylation levels for watered and dehydration-stressed conditions, peak calls, our gene expression results, exons, UTRs, alternative transcription start sites are displayed in the implementation of the UC Santa Cruz Genome Browser [13] at the Bioinformatics Core Facility at the University of Nebraska-Lincoln. Exons, transcription start sites and untranslated regions were downloaded from the TAIR [11]ftp://ftp.Arabidopsis.org/home/tair/Genes/. The results can be accessed at: http://bioinformatics.unl.edu/h3k4me/cgi-bin/hgGateway. The ChIP-Seq data has been deposited at the NCBI Gene Expression Omnibus under series record GSE11657 (part of SuperSeries record GSE11658).

## Results

### Changes in gene expression in plants exposed to soil water deficit conditions

Plants were watered or subjected to dehydration stress by withholding water until a relative water content (RWC) of 65% was reached. A RWC of 65% causes leaf wilting and is considered a moderate level of dehydration stress [14]. The mRNAs from rosette leaves of non-stressed (control) and dehydration-stressed Arabidopsis plants were analyzed by microarrays (Additional file 2, Table S1). Further analysis indicated 640 genes increased, and 525 genes decreased, their transcript levels by 4-fold or more (*p *≤ 0.001) as result of the dehydration stress (Additional file 3, Table S2). Analysis of the most highly represented gene categories (using the Gene Ontology classification) indicated that the majority of the genes with altered expression was involved in various stress-responses (Additional file 4, Table S3). For example, within the list of genes responding to dehydration-stress, many genes have been also recognized as components of pathways stimulated by cold, temperature, light, and radiation (~750 genes), by hormones, endogenous, and chemical stimuli (~300 genes), as well as genes encoding transcription factors and regulators (131 genes). Genes for metabolic enzymes, and for oxidoreductase functions, were also affected (Additional file 4, Table S3). Among the genes up-regulated by dehydration stress, well-known genes implicated in this response in other studies were present, including genes ABF3 (ABA responsive element binding factor 3, *AT4G34000*, [15]), CBF4 (C-repeat binding factor 4, *AT5G51990 *[16]), RD26 (Responsive to desiccation 26, *AT4G27410*, [17]), RD29A (*AT5G52310*), RD29B (*AT5G52300*), ATHB7 (*Arabidopsis thaliana *homeobox 7, *AT2G46680*) and ATHB12 (*AT3G61890*; [18]).

### Genome-wide distributions of H3 and mono-, di-, and tri-methylated H3K4 in watered and in dehydration stressed plants

**Figure 1:**
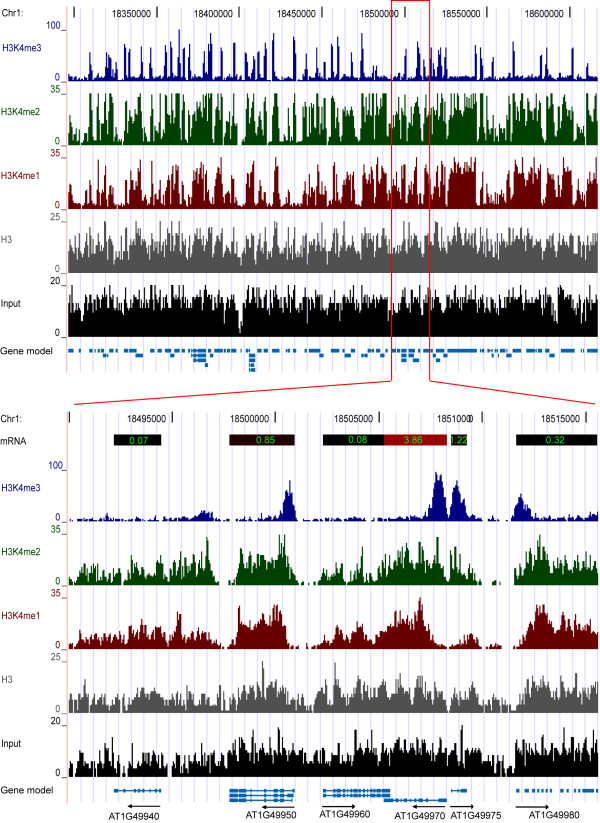
**UCSC browser view of genomic data of a representative chromosomal region**. A representative region of chromosome 1 representing 250,000 bp and an expanded region of 20,000 bp are shown. For simplicity, only data from samples from the watered condition are shown. The chromosomal coordinates are shown at the top of each region. The amount of sequence coverage for the different samples is shown to the left of each track (y-axis), and represents the number of times each region was recovered by sequencing. The tracks are: tri-methylated H3K4 (H3K4Me3, blue) di- methylated H3K4 (H3K4Me2, green), and mono-methylated H3K4 (H3K4Me1, red), total H3 (H3, gray), and total input DNA (Input, black). The magnified section shows the mRNA levels at the top as a color scale with the values shown in each colored bar. The gene models below indicate the gene name and direction.

To examine whether and how chromatin reacted during the stress response at the whole-genome level, we used ChIP-Seq to analyze the changes in the global chromatin methylation marks on H3K4 between the watered and dehydration stressed samples (see Additional file 5, Table S4 for the number of sequences analyzed for each sample). The ChIP-Seq chromatin data was visualized using a local implementation of the UCSC Genome Browser [19]. A representative chromosomal region demonstrating the types of coverage obtained from total input DNA, and from antibodies to total H3 or specific to mono-, di- or tri-methylated H3K4 is shown (Figure 1). The antibodies for the H3K4me1, H3K4me2, and H3K4me3 forms of H3 were analyzed for their ability to recognize a panel of peptides containing these modifications. The antibodies to H3K4me1 and H3K4me3 were highly specific, while the antibody to H3K4me2 had weak recognition of H3K4me3 (Additional file 1, Figure S1). This weak cross reactivity did not appear to affect the ChIP-SEQ results as the profile of H3K4me2 was very different than the profile of H3K4me3 (Figure 1). Samples containing total input DNA (not subjected to chromatin immunoprecipitation), or DNA from samples immunoprecipitated with antibody that recognized H3 regardless of its methylation state, did not show the gene-centric enrichment identified by the antibodies to the methylated forms of H3K4 (Figure 1). The distributions of the methylated forms of H3K4 were further analyzed below. The accuracy of the ChIP-Seq method was verified by quantitative PCR (qPCR) of arbitrarily selected gene regions (Additional file 6, Table S5) and a comparison of the data sets indicated they had a good quantitative agreement, with a correlation coefficient of 0.7.

A detailed analysis of the genomic locations of the peaks of H3K4 methylation indicated that the majority of the peaks mapped to genes. The percentage of the peaks for the individual mono-, di-, or tri-methylated H3K4 types that were located on gene regions ranged from 80% to 88% of the total peaks (Additional file 7, Table S6). Although the majority of peaks mapped to genes, not all genes contained each type of H3K4 methylation: the percentage of genes containing mono-, di-, or tri-methylation of H3K4 ranged from 62 to 84% (Table 1). 9.9% of annotated genes did not contain any form of H3K4 methylation (Table 1). The majority of the genes lacking any type of H3K4 methylation were in the two lowest expression quintiles (data not shown).

**Table 1:** Percentage of genes with H3K4 methylation regions^1^

*Treatment and type of H3K4 methylation*	*Number of genes*	*Percentage of genes*
*Water: H3K4me1*	21875	80.5
*Dry: H3K4me1*	22167	81.6
*Water: H3K4me2*	22708	83.6
*Dry: H3K4me2*	22953	84.5
*Water: H3K4me3*	16769	61.7
*Dry: H3K4me3*	18072	66.5
*Genes with one or more types of H3K4 methylation*	24487	90.1

*Genes lacking H3K4 methylation*	2682	9.9

^1^H3K4 methylation region analyzed includes 200 bp upstream and downstream of the transcribed regions of 27,169 annotated genes.

### Genome-wide distribution of histone H3, H3K4me1, me2 and me3 at gene sequences: correlation with transcription activity

**Figure 2:**
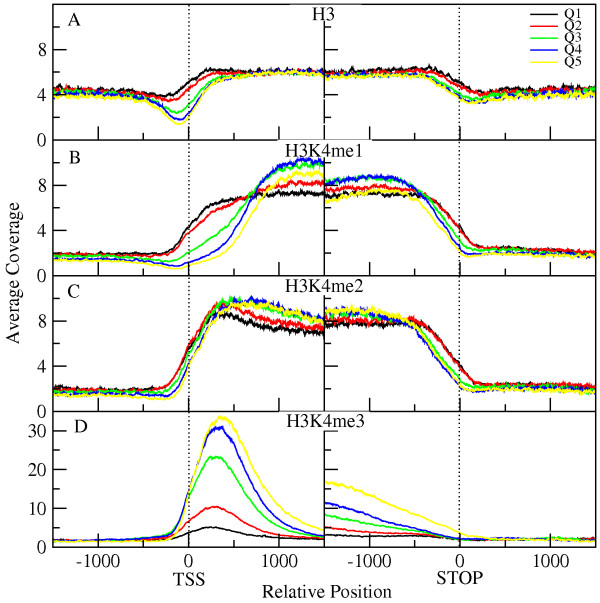
**H3K4 methylation profiles of genes with different transcript levels**. Genes were sorted into quintiles according to their transcript levels. These sets are indicated by the different colors shown in the insert box at the top of the Figure, with yellow (Q5) representing the 20% of genes with the most abundant transcripts. Total H3 or the type of H3K4 methylation measured is indicated above each graph. The *y*-axis of each graph shows the amount of H3 or H3K4 methylation after normalization (using the input DNA coverage profile) as measured by the number of times that region was detected by sequencing (Average Coverage). The *x*-axis shows the gene position in bp relative to either the transcription start site (TSS on left side of the Figure) or stop codon (on right side of the Figure) of the genes.

Next, we examined how overall gene transcription activity was associated with H3 or H3K4 methylation levels on genes. Genes were grouped into expression quintiles based on their transcript levels. The amount of H3 or mono-, di-, or tri-methylated H3K4 was plotted at their 5'- and 3'-ends (Figure 2). We observed that histone H3 occupancy was slightly enriched within genes (Figure 2A). There was a significant dip in the H3 (nucleosome) occupancy immediately 5' of the transcription start site (TSS) for the highly expressed genes (the top three quintiles, Figure 2A). This region could correspond to a nucleosome free region (NRF) that occurs at actively transcribed genes [20]. At the 3'-end of the genes, this transcriptional effect on H3 levels was much less pronounced.

The profiles of mono-, di-, and tri-methylated H3K4 displayed specific patterns associated with transcript levels. H3K4me1 levels remained fairly constant throughout genes (Figure 2B). One pattern of interest was that the 5'-regions of the genes with the lowest expression levels had higher H3K4me1 levels than more highly expressed genes (Figure 2B). The profiles of H3K4me2 distribution had no correlation with transcript levels, and were relatively constant throughout the transcribed regions (Figure 2C). As such, H3K4me2 marked 84% of genes (Table 1) irrespective of the transcript levels produced from these genes. Both H3K4me1 and H3K4me2 were fairly constant across the middle and end of the genes, and accurately marked the ends of the genes.

In contrast, the H3K4me3 distribution showed a clear peak around 300 bp downstream of the TSS. However, the magnitudes of signal values for the five expression-based quintiles were in reverse to those displayed by H3K4me1: the highest H3K4me3 peaks were for genes with the highest transcript levels. The H3K4me3 signal at the 5'-ends of the least actively transcribed genes displayed the lowest coverage values (Figure 2D). Characteristically, the peak of H3K4me3 distribution tapers off towards the 3' end of the genes and reaches intergenic levels at the 3'-gene ends.

### H3K4 methylation changes when transcript levels change

**Figure 3:**
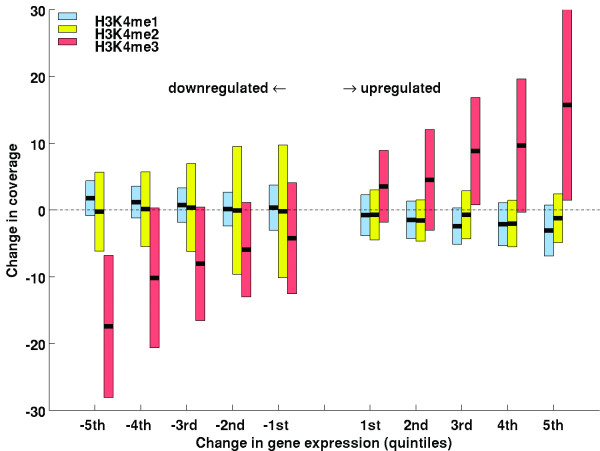
**Changes in H3K4 methylation for genes with changes in mRNA levels**. Genes that were up- or down-regulated by dehydration stress were grouped into up- or down-regulated quintiles according to their change in transcript levels. The genes showing the largest increase or largest decrease in transcript levels were placed into the 5^th ^or -5^th ^quintiles, respectively. The type of H3K4 methylation is represented by the color key in the upper left corner of the chart. The change in the amount of H3K4 methylation for each type of H3K4 methylation is represented on the *y*-axis (Change in coverage). For example, a median value (black cross bar) showing no change in H3K4 methylation would be at *y*-coordinate 0 and a median value showing an increase of 15 in coverage would be placed at *y*-coordinate + 15. One standard deviation in H3K4 methylation coverage is shown on each side of the median value.

To determine whether/how stress-altered gene expression correlated with changes in H3K4 methylation, we examined genes whose transcripts increased or decreased by 4-fold or more, and had highly significant *p*-values (p ≤ 0.001; Additional file 3, Table S2). The 1165 genes meeting these criteria were sorted into five up-regulated and five down-regulated change-of-expression quintiles, i.e., the genes with the biggest increase or decrease in transcript levels were in the highest (5^th^) up or down regulated change-of-expression quintile, respectively. The median and range of changes in H3K4me1, me2, or me3 methylation amounts were plotted for each of the up- and down-regulated expression quintiles (Figure 3). The median H3K4me1 levels slightly decreased as the magnitude of the up-regulated transcript changes increased and slightly increased as the magnitude of the transcript reductions increased. Thus, H3K4me1 levels were inversely or negatively associated with changes in transcript levels. The median for the H3K4me2 values slightly decreased for up-regulated quintiles, and was little changed for down-regulated quintiles (Figure 3).

The most significant changes were displayed by the H3K4 tri-methylation profiles. The median and the majority of the genes within one standard deviation of the median increased for all up-regulated quintiles and decreased for all down regulated quintiles (Figure 3). In addition, the largest increase in H3K4me3 levels was found on nucleosomes of the genes in the highest expression quintile, indicating that the magnitude of the H3K4me3 increase was positively associated with increased transcript levels. Similarly, the down-regulated quintile with the largest decrease in transcript levels had the largest decrease in H3K4me3 levels (Figure 3).

As a control for our method of analysis, we analyzed genes that did not change transcript levels and found that these genes did not show significant variation in their H3K4 methylation levels. Correlation coefficients of 0.95, 0.88, and 0.93 were observed between the watered and dehydration-stressed samples for the H3K4 mono-, di-, or tri-methylation comparisons of genes that did not change transcript levels, respectively. These high correlation coefficients indicated that there was very little change in H3K4 methylation in this set of genes (Additional file 8, Figure S2).

### H3K4 methylation profiles of selected genes during watered or dehydration stressed conditions

**Figure 4:**
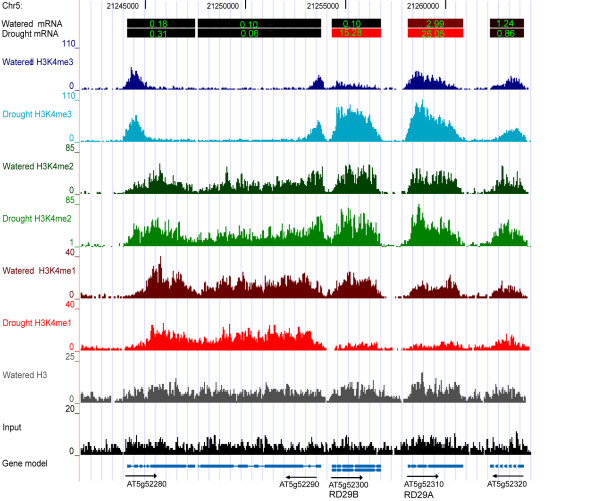
**Genome browser view of the chromosomal region containing RD29A and RD29B**. A region of chromosome 5, with the indicated position numbers, containing the dehydration and ABA inducible RD29A and RD29B genes as well as three flanking genes is shown. Arrows at the bottom show the direction of transcription below the representation of each gene. The transcript levels of the watered or dehydration stressed samples are shown as colored bars above each gene region, with the relative transcript levels shown inside each colored bar. The type of treatment and what was measured (mRNA levels, type of H3K4 methylation, total H3, or input DNA) are shown at the left. The histogram bars measure the number of times each region was identified by sequencing (y-axis coverage scale with a top value range of 20 to 110, depending on the coverage range of each sample).

A specific chromosomal region of interest demonstrating changes in H3K4 methylation status after dehydration stress is the region containing the ABA and dehydration-induced genes *RD29A *(*AT5G52310*) and *RD29B *(*AT5G52300*, Figure 4). Microarray data indicated that transcript levels of *RD29A *and *RD29B *were induced 9 and 147 fold by dehydration stress, respectively (transcript levels are shown as a color scale with insert values in Figure 4). The neighboring genes (AT5G52280, *AT5G52290, and AT5G52320*) did not change their expression (color scale at top of Figure 4) or H3K4 methylation levels. *RD29A *had a higher basal transcription rate than *RD29B *in the watered control treatment and had a higher basal level of H3K4me3. Their expression increased during dehydration stress as did the H3K4me3 levels on the nucleosomes of both genes. There was a corresponding decrease in H3K4me1 levels while H3K4me2 levels showed little change.

Similar tendencies were demonstrated by other dehydration stress responsive genes as well. Six representative chromosomal regions, representing genes that were induced, repressed, or unaffected by dehydration stress conditions, are shown in a UCSC Genome Browser view (Additional file 9, Figure S3). For induced genes, the H3K4me3 levels increased during dehydration stress as their expression increased, with a corresponding decrease in H3K4me1, while H3K4me2 levels showed little change (Additional file 9, Figure S3A, S3B). The two dehydration-stress repressed genes showed decreased H3K4me3 levels, and a slight increase in H3K4me1 (Additional file 9, Figure S3C and additional file 10, Figure S3D). By comparison, genes without changes in gene expression did not display significant changes in any of the H3K4 methylation-forms (Additional file 10, Figure S3E, S3F). Collectively, the changes, or lack of changes, in the H3K4 methylation patterns displayed at individual gene loci were consistent with the overall genomic patterns as described above.

### **Dehydration stress responsive genes show broader H3K4me3 distribution profiles**

**Figure 5:**
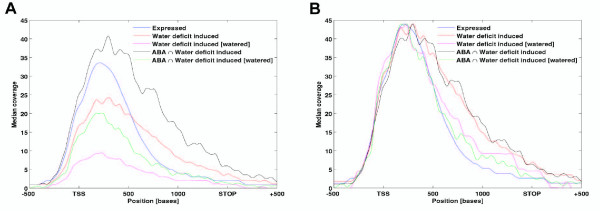
**Dehydration induced genes show broader regions of H3K4me3**. The median value of H3K4me3 coverage was calculated at positions along the genes for three different sets of genes. Each set was further limited to contain only genes between 1,000 to 2,000 bp in length. The three sets of genes were: all expressed genes, all dehydration induced genes, and all dehydration induced genes also known to be ABA regulated. These groups are indicated by the color key at upper right of the Figure. The median level of H3K4me3 observed for each set of genes (*y*-axis, Median coverage) was determined at different positions along the genes (*x*-axis in bp, with genes normalized to a standard 1500 bp size between the TSS and STOP codon and containing 500 bp upstream or downstream from these sites). **(A) **The actual coverage profiles without peak normalization. **(B) **Profiles that were normalized for peak heights to facilitate comparison of the profile shapes. The *p*-values for the normalized profiles for the dehydration/ABA induced genes in **(B)**, which measure the probability of the broader profiles occurring by chance, were *p *= 0.002 for the watered condition and *p *= 1.4 × 10^-10 ^for the water deficit condition.

Inspection of individual dehydration-stress induced genes indicated the H3K4me3 distribution at these genes tended to be distributed over longer gene regions when compared to the H3K4me3 patterns at unresponsive genes (see Figure 4). We then asked how general this broader H3K4me3 distribution pattern was for induced genes. To accomplish this, we measured the profiles of H3K4me3 modification along the length of three groups of genes: all expressed genes, dehydration-induced genes, and the subset of dehydration induced genes that were also ABA induced [21]. The median values of H3K4me3 modification along the length of these genes were then plotted to a gene length normalized to 1500 bp. These profiles were plotted with their actual median coverage levels or with each peak normalized to the same maximum level for a better relative comparison (Figure 5). The profiles for the dehydration or dehydration/ABA induced genes are also shown for the watered condition for these genes as well (Figure 5). All five curves of H3K4me3 levels for the three sets of genes had peaks about 300 bp downstream of the TSS and then progressively declined towards the stop codon of the genes. However, the profile for all expressed genes showed a faster decrease in H3K4me3 levels, while the dehydration-induced or subset of known ABA-induced genes demonstrated a broader H3K4me3 distribution pattern (Figure 5B). This broader distribution of H3K4me3 was significant for the dehydration/ABA induced genes (p = 1.4 × 10^-10^). Surprisingly, this broad H3K4me3 distribution was also significant (p = 0.002) for these genes in the watered condition prior to dehydration stress. This suggests that some biological feature of the dehydration/ABA induced genes causes these broader than normal H3K4me3 distributions in both the watered and dehydration/ABA induced states.

## Discussion

Here we report the first whole genome map of the histone H3K4 methylation patterns of chromatin during a plant's response to dehydration stress. Using the ChIP-Seq approach, we found that the distribution of mono-, di-, and tri-methylation of H3K4 in Arabidopsis were predominantly located on genes, in general agreement with prior studies [22]. However, in watered plants we identified 21,875 genes carrying H3K4me1 *versus *15,475 genes found by the ChIP-chip method; 22,708 genes with the H3K4me2 mark *versus *12,781, and 16,769 H3K4me3-tagged genes *versus *15,894 genes reported in [22]. Furthermore, our results indicated that only 10% of expressed genes did not contain at least one H3K4me type, while ~1/3 of the genes was found to be free of H3K4 methylation by the ChIP-chip approach. These differences might reflect different sensitivities of the two methods and/or different tissue distributions in the whole seedlings [22]*versus *rosette leaves analyzed (this study). The gene-associated location of H3K4me modifications did not change during the dehydration stress-responding process. Within this gene-centric framework, however, the abundances of the individual forms of H3K4 methylation displayed specific profile changes associated with altered transcription from these genes.

### H3 distribution and nucleosome free regions

The genome-wide distribution of histone H3 was used to gain insight to whether a region lacked nucleosomes or lacked H3K4 methylation of those nucleosomes. The H3 distribution showed a fairly continuous presence across genic and intergenic regions. By contrast, the H3K4 methylated forms were practically absent from intergenic regions. In agreement with observations made in yeast [23] and Arabidopsis [24], we also observed an H3 enrichment in gene regions relative to intergenic regions. Furthermore, in agreement with other genomic analyses in Arabidopsis [24-26], our analysis of the H3 profiles in genes grouped by transcript levels, revealed regions with low H3 presence, "nucleosome free regions" (NFRs), identified immediately upstream of the TSS for more highly expressed genes. NFRs have also been observed in yeast [27-29], *Drosophila *[30], and human cells [31]. Interestingly, nucleosome density has also been observed to be different in exon and intron gene regions [32]. It has been suggested that the nucleosome occupancy is influenced by the DNA sequence, which might facilitate nucleosome removal upon transcription [33]. Our finding that NFRs are associated with transcriptionally active genes is consistent with the idea of nucleosome displacement by transcription factors [27] or complexes involved in transcription [29]. Similarly, transcription from the Arabidopsis *AP1*, a gene required for the initiation of flowering, requires the reprogramming of the *AP1 *locus to an actively transcribed state that is accompanied by the removal of a nucleosome from the transcription start site and its dynamic re-positioning in a developmentally regulated process [34].

### H3K4me1 distribution changes

The majority of genes with increased transcript levels showed modest reductions in H3K4me1 levels. Conversely, the most strongly down-regulated genes showed an increase in H3K4me1 marks, while genes with smaller reductions in transcript levels did not detectably change their average H3K4me1 levels. A negative association of H3K4me1 levels with increased transcription might be due to conversion to the more abundant H3K4me2 and H3K4me3 forms at higher transcription rates. Alternatively, higher H3K4me1 levels, particularly near the TSS, might repress transcription. A repressive role has been reported in *Chlamydomonas *where H3K4 mono-methylation was found associated with repressed transgenes and transposable elements. The loss of this mark in *Chlamydomonas *mutant *Mut11 *resulted in expression of these genes [35]. The possible role of H3K4me1 in gene repression might be unique to the plant kingdom as H3K4me1 levels are associated with enhancers in animal cells [36].

### H3K4me2 distribution changes

In our studies, 84% of Arabidopsis genes carried H3K4me2 marks, and on average, these marks were distributed relatively uniformly along gene sequences. In agreement with an earlier genomic study [22], H3K4me2 levels were not associated with transcript levels in watered plants. In response to dehydration stress, H3K4me2 levels were only slightly reduced when transcript levels increased, and showed little change when transcript levels were reduced. Previous ChIP-PCR analysis of individual genes in Arabidopsis found that H3K4me2 marked 5' boundaries of the tested genes regardless of expression levels [37]. These observations suggest H3K4me2 marks do not correlate with transcript levels and may play a role in gene regulation different from the roles of mono-, and tri-methylated nucleosomes.

### H3K4me3 distribution changes

H3K4me3 modification was predominantly associated with actively transcribed genes. Our observation that higher H3K4me3 levels were associated with higher transcript levels was in agreement with studies in yeast [38], rice [39], Arabidopsis [22,24], and human cells [40,41]. This tendency was dynamic, as we found up-regulated genes had increased H3K4me3 levels, and down-regulated genes had reduced H3K4me3 levels. These findings are in agreement with numerous results from individual plant genes, where H3K4me3 was found to increase with increased transcription at dehydration induced promoters [6], or the phaseolin [42], alcohol dehydrogenase 1 (*ADH1*) and pyruvate decarboxylase 1 (*PDC1*) genes [4]. The magnitude of the change, however, only weakly correlated with the magnitude of the change in transcript levels as shown by the wide range of H3K4me3 values in each expression quintile (Figure 3).

The genome-wide H3K4me3 distribution in Arabidopsis displays a peak at +300 bp relative to the TSS (Figure 2D, [22]), similar to the location observed in human cells [40], but slightly upstream of the peak location in rice observed at about +500 bp [39]. A surprising deviation from this pattern was the broad H3K4me3 distribution profiles present on many dehydration/ABA inducible genes. Interestingly, this broad pattern was present on many of these genes prior to their induction, although the lower H3K4me3 levels made this pattern less striking than in the induced state. For example, both *RD29A *and *RD29B *had low levels of H3K4me3 in a broad distribution pattern that was similar to the broad pattern of the higher levels of H3K4me3 observed during dehydration stress (Figure 4). Although the occurrence of this pattern was statistically significant, its functional significance is unclear.

## Conclusions

Although there is a strong association of H3K4me3 with transcriptionally active genes, the biochemical roles of H3K4me3 are emerging as occurring predominantly after transcription initiation [43-45]. H3K4me3 marks are recognized by chromatin remodeling factors facilitating transcription by altering the structure, composition, and positioning of nucleosomes, by components of the spliceosome, and by proteins involved in mRNA capping and stability [46]. Recognition and binding to H3K4me3 has been traced to plant homeodomains or chromodomains present in these proteins [47]. Additionally, in support of a post-transcriptional function, the yeast SET1 H3K4 methyltransferase responsible for H3K4me3 modification is recruited to the +300 bp region of genes after transcription initiation in yeast [48]. Elevated H3K4me3 levels occurring on genes with increased transcript production might effectively recruit more chromosomal and RNA processing proteins, thereby facilitating the increased transcriptional activity on these induced genes.

## Authors' contributions

KVD, YD, HC, and YX designed and performed the experiments; SM, JJMR, RL, JY, PL, and IL computationally analyzed the data; ZA and MF wrote the manuscript. All authors read and approved the final manuscript.

## Supplementary Material

Additional File 1 **Figure S1. Specificity of the H3K4 methylation antibodies**. The antibodies to H3K4me1, H3K4me2, or H3K4me3 were tested against a panel of peptides containing or lacking these modifications. Click here for file

Additional File 2 **Table S1. Microarray analysis of dehydration-stressed Arabidopsis plants**. Arabidopsis plants were watered or subjected to dehydration stress to a relative water content of 65% prior to RNA isolation and microarray analysis. RNA levels before and during dehydration stress are shown. Click here for file

Additional File 3 **Table S2. Dehydration responsive genes that change by at least 4-fold**. A subset of genes derived from Table S1 that change expression levels by at least 4-fold. Click here for file

Additional File 4 **Table S3. Gene Ontology terms for the top categories of up or down regulated genes**. The genes derived from Table S2 were classified by their gene ontology terms. Click here for file

Additional File 5 **Table S4. Number of sequencing reads from each chromatin immunoprecipitation experiment**. The number of sequencing reads analyzed in the ChIP-Seq or input DNA-SEQ experiments is shown. Click here for file

Additional File 6 **Table S5. Quantitative comparison of high-throughput sequencing and qPCR measurements of ChIP samples**. The magnitude of the changes in ChIP of H3K4me3 levels recovered in the watered or dehydration-stressed samples was measured for selected regions by ChIP-SEQ and real time PCR. The sequences of the primers used are also provided. Click here for file

Additional File 7 **Table S6. Genomic location of regions enriched for H3K4 methylation**. The intergenic or intragenic locations of the peaks of H3K4me3 were determined across the genome for mono-, di-, and tri-methylated H3K4 in the watered and dehydration-stressed conditions. Click here for file

Additional File 8 **Figure S2. Analysis of changes in H3K4 methylation types for genes that did not change expression when stressed**. The levels of H3K4 mono-, di-, or tri-methylation for genes that did not show changes in transcript levels were plotted for the watered vs dehydration stress condition. Click here for file

Additional File 9 **Figure S3 A-C. Genome browser view of the chromosomal region genes with up- or down-regulated expression levels**. The genome browser view of H3K4 mono-, di-, or tri-methylation levels for genes with increased or decreased gene expression are shown. Click here for file

Additional File 10 **Figure S3 D-F. Genome browser view of the chromosomal region genes with down- regulated or unchanged expression levels**. The genome browser view of H3K4 mono-, di-, or tri-methylation levels for genes with decreased or no change in gene expression are shown. Click here for file
