# Supplementary material for: Dynamic changes in genome-wide histone H3 lysine 4 methylation patterns in response to dehydration stress in Arabidopsis thaliana
Source: BMC Plant Biol. 2010 Nov 5;10:238. doi: 10.1186/1471-2229-10-238 (PMC3095321; doi:10.1186/1471-2229-10-238)
Supplement: Additional File 1 — Figure S1. Specificity of the H3K4 methylation antibodies. The antibodies to H3K4me1, H3K4me2, or H3K4me3 were tested against a panel of peptides containing or lacking these modifications. [file 1471-2229-10-238-S1.DOC]

Additional File 5 Figure S1


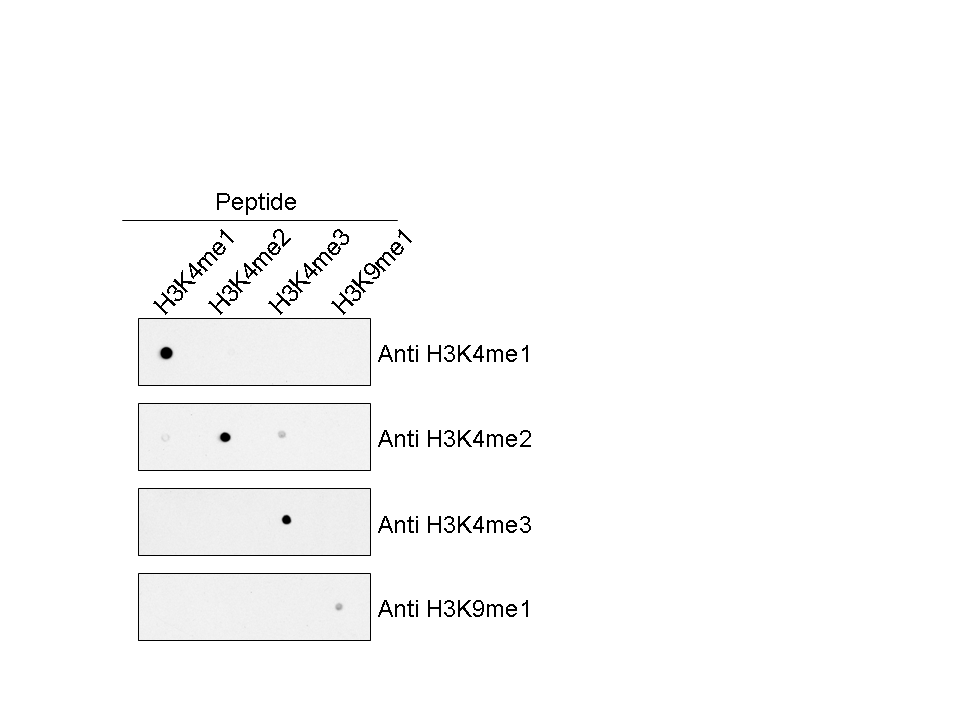


**Additional File 5** **Figure S1.** 50 ng of peptides derived from the first 100 amino acids of the N-terminal sequence of human H3 and containing one or more methyl groups at lysine 4: H3K4me1 (abcam ab1340), H3K4me2 (abcam ab7768), H3K4me3 (abcam ab1342), or lysine 9: H3K9me1 (abcam ab1771) were spotted on a PVDF membrane and probed with the indicated antibodies.
