# Supplementary material for: Dynamic changes in genome-wide histone H3 lysine 4 methylation patterns in response to dehydration stress in Arabidopsis thaliana
Source: BMC Plant Biol. 2010 Nov 5;10:238. doi: 10.1186/1471-2229-10-238 (PMC3095321; doi:10.1186/1471-2229-10-238)
Supplement: Additional File 4 — Table S3. Gene Ontology terms for the top categories of up or down regulated genes. The genes derived from Table S2 were classified by their gene ontology terms. [file 1471-2229-10-238-S4.DOC]

Additional File 3 Table S3. GO terms for top categories of genes up- or down-regulated by dehydration stress.

| Up-regulated | | |  | Down-regulated | | |
| --- | --- | --- | --- | --- | --- | --- |
| GO term | *P*-value | Genes |  | GO term | *P-*value | Genes |
|  |  |  |  |  |  |  |
| response to water | < 10^-27^ | 36 |  | response to stimulus | < 10^-13^ | 110 |
| response to water deprivation | < 10-^26^ | 34 |  | catalytic activity | < 10^-11^ | 168 |
| response to abscisic acid stimulus | < 10^-23^ | 38 |  | anchored to membrane | < 10^-11^ | 26 |
| response to abiotic stimulus | < 10^-22^ | 79 |  | plant-type cell wall | < 10^-10^ | 25 |
| response to endogenous stimulus | < 10-^17^ | 58 |  | intrinsic to membrane | < 10^-9^ | 28 |
| response to stimulus | < 10^-17^ | 136 |  | response to chemical stimulus | < 10^-8^ | 59 |
| response to stress | < 10^-16^ | 89 |  | response to hormone stimulus | < 10^-8^ | 35 |
| response to hormone stimulus | < 10^-16^ | 53 |  | response to chitin | < 10^-7^ | 14 |
| response to chemical stimulus | < 10^-16^ | 85 |  | response to endogenous stimulus | < 10^-7^ | 36 |
| catalytic activity | < 10^-14^ | 204 |  | hydrolase activity | < 10^-7^ | 69 |
| response to temperature stimulus | < 10^-12^ | 32 |  | biological regulation | < 10^-6^ | 76 |
| secondary metabolic process | < 10^-9^ | 28 |  | protein amino acid phosphorylation | < 10^-6^ | 18 |
| response to heat | < 10^-9^ | 16 |  | hydrolase activity, acting on glycosyl bonds | < 10^-6^ | 22 |
| response to osmotic stress | < 10^-9^ | 30 |  | carboxylesterase activity | < 10^-5^ | 16 |
| oxidoreductase activity | < 10^-8^ | 45 |  | response to carbohydrate stimulus | < 10^-5^ | 14 |
| UDP-glycosyltransferase activity | < 10^-7^ | 18 |  | transferase activity | < 10^-5^ | 68 |
| transcription factor activity | < 10^-7^ | 65 |  | response to organic substance | < 10^-5^ | 14 |
| transferase activity, transferring hexosyl groups | < 10^-6^ | 19 |  | response to light stimulus | < 10^-5^ | 21 |
| transferase activity, transferring glycosyl groups | < 10^-6^ | 26 |  | phosphorylation | < 10^-5^ | 18 |
| transcription regulator activity | < 10^-6^ | 66 |  | response to radiation | < 10^-5^ | 21 |
