# Supplementary material for: Dynamic changes in genome-wide histone H3 lysine 4 methylation patterns in response to dehydration stress in Arabidopsis thaliana
Source: BMC Plant Biol. 2010 Nov 5;10:238. doi: 10.1186/1471-2229-10-238 (PMC3095321; doi:10.1186/1471-2229-10-238)
Supplement: Additional File 5 — Table S4. Number of sequencing reads from each chromatin immunoprecipitation experiment. The number of sequencing reads analyzed in the ChIP-Seq or input DNA-SEQ experiments is shown. [file 1471-2229-10-238-S5.DOC]

Additional File 4 Table S4. Number of sequencing reads from each chromatin immunoprecipitation experiment

| *Antibody* | *^a^Number of sequencing reads* | |
| --- | --- | --- |
|  | *Watered* | *Not Watered* |
| H3K4me1 | 17,451,837 | 16,972,749 |
| H3K4me2 | 27,354,179 | 39,299,903 |
| H3K4me3 | 12,285,745 | 18,012,924 |
| H3 | 11,269,371 |  |
| Input DNA | 8,862,048 |  |

**^a^**Number of sequences that are unique in the Arabidopsis genome and contain 2 or less mismatches
