# Supplementary material for: Dynamic changes in genome-wide histone H3 lysine 4 methylation patterns in response to dehydration stress in Arabidopsis thaliana
Source: BMC Plant Biol. 2010 Nov 5;10:238. doi: 10.1186/1471-2229-10-238 (PMC3095321; doi:10.1186/1471-2229-10-238)
Supplement: Additional File 6 — Table S5. Quantitative comparison of high-throughput sequencing and qPCR measurements of ChIP samples. The magnitude of the changes in ChIP of H3K4me3 levels recovered in the watered or dehydration-stressed samples was measured for selected regions by ChIP-SEQ and real time PCR. The sequences of the primers used are also provided. [file 1471-2229-10-238-S6.DOC]

Additional File 6 Table S5. Quantitative comparison of high-throughput sequencing and qPCR measurements of ChIP samples^1^

| **Gene** | **TAIR #** | **Gene Response to water deficit stress** | **Method used to measure H3K4me3 levels** | | | | |
| --- | --- | --- | --- | --- | --- | --- | --- |
|  |  |  | **Illumina watered coverage** | **Illumina drought coverage** | **Illumina fold change** | **Q-PCR fold change** | **Q-PCR standard deviation** |
| ***GAPC2*** | AT1G13440 | Constitutive | 208 | 109 | 0.5 | 0.7 | 0.12 |
| ***eEF1b2*** | AT5G19510 | Constitutive | 144 | 244 | 1.7 | 1.6 | 0.23 |
| ***RAB18*** | AT5G66400 | Induced | 155 | 4229 | 27.3 | 10.3 | 0.57 |
| ***CBF4*** | AT5G51990 | Induced | 365 | 1976 | 5.4 | 2.7 | 0.17 |
| ***XERO2*** | AT3G50970 | Induced | 325 | 2094 | 6.4 | 4.6 | 0.14 |
| ***ATHB7*** | AT2G46680 | Induced | 442 | 5574 | 12.6 | 4.5 | 0.38 |
| ***ATHB12*** | AT3G61890 | Induced | 3474 | 8670 | 2.5 | 1.6 | 0.13 |
| ***SAG29*** | AT5G13170 | Induced | 243 | 2146 | 8.8 | 13.8 | 2 |
| ***RD29B*** | AT5G52300 | Induced | 851 | 3766 | 4.4 | 2.4 | 0.7 |
| ***RD29A*** | AT5G52310 | Induced | 6295 | 14096 | 2.2 | 1.7 | 0.6 |
| ***LR4 (LTP4)*** | AT5G59310 | Induced | 330 | 2317 | 7.0 | 2.0 | 0.5 |
| ***GLP1*** | AT1G72610 | Repressed | 5390 | 1990 | 0.4 | 0.5 | 0.1 |
| ***LTP*** | AT2G10940 | Repressed | 1381 | 433 | 0.3 | 0.4 | 0.1 |

^1^The primer sequences of genes analyzed by real time PCR are shown in the Supplementary Table below. The DNA levels were normalized to ACT7 (AT5G09810). Experiments were repeated two times, and each sample was analyzed using three replicates of each DNA pool, and the relative amounts of each target DNA and ACT7 were calculated using the comparative C_T_ method [according to the manufacturer’s software (Bio-Rad, Hercules, CA)]. Cycle numbers were used to calculate gene expression levels in the linear amplification range.

Primers Used

| **TAIR gene number** | **The amplifcation region** | **Primer Name** | **Primer Sequence(5'-3')** |
| --- | --- | --- | --- |
| AT3G61890 | chr3:22925457-22925595 | ATHB12 Set 1 Forward Primer | TGA TCA GTC TCT TCC TCG AAC CCA |
|  |  | ATHB12 Set 1 Reverse Primer | TAA GCA GCA GCA CAG AGT CGC ATA |
| AT5G51990 | chr5:21134569-21134649 | CBF4 Set 1 Forward Primer | TTT AGA TCC CTC CGT CGT AGT CTC |
|  |  | CBF4 Set 1 Reverse Primer | CTT GTC CTA AGG AGA TTC AGA AAG CTG CG |
| AT5G66400 | chr5:26535656-26535748 | RAB18 Set 1 Forward Primer | GCA CAA TAC AAC GAC CGA ATG CGA |
|  |  | RAB18 Set 1 Reverse Primer | GGT GGC CGT TAA GCT TCG AAC AAT |
| AT1G13440 | chr1:4608203-4608342 | GAPC2 Good Set 1 Forward Prime | TGT TCC AGT TGC CAG TTG GGT TTG |
|  |  | GAPC2 Good Set 1 Reverse Primer | CCA TGC GCA GTC ATG AGA GTT TGT |
| AT3G50970 | chr3:18952175-18952277 | XERO2 Set 1 Forward Primer | ACT GGG ACT AAC ACG GCT TAT GGT |
|  |  | XERO2 Set 1 Reverse Primer | TTC CAT GAT GAC CTG GCA GTT GCT |
| AT2G46680 | chr2:19172858-19173006 | ATHB7 Set 1 Forward Primer | AAT CCC GCC AAG GGT AAT TGT TGC |
|  |  | ATHB7 Set 1 Reverse Primer | TGG TGG GTT TGA GGA AGA ACC AGA |
| AT2G10940 | chr2:4317740-4317840 | LTP Set 3 Forward Primer | ACA AGC ATA AGG GAA CGA TAG AGG |
|  |  | LTP Set 3 Reverse Primer | GAG AAG AGA ATG GCA GAG CTC CAA |
| AT5G19510 | chr5:6582140-6582231 | eEF1b2 Set 1 Forward Primer | ATC TCA ACA CCA CGA ACA GCC TCT |
|  |  | eEF1b2 Set 1 Reverse Primer | GGT GGA AAG TCT TCT GTG CTC ATG GA |
| AT5G09810 | chr5:3052153-3052286 | Actin ACT7 P112 | P112 cgtttcgctttccttagtgttagct |
|  |  | Actin ACT7 P113 | P113 agcgaacggatctagagactcac |
| AT5G52300 | chr5:21254345-21254489 | RD29B P114 | P114 cagagacacgaaaagaaagaaaacaac |
|  |  | RD29B P115 | P115 attctgattggttcttctgcttgc |
| AT5G52310 | chr5:21258564-21258716 | RD29A P132 | P132 gcaggcgtaacaggtaaac |
|  |  | RD29A P133 | P133 gcatcgtgtccgtaagagg |
| AT5G13170 | chr5:4183052-4183284 | Sag29 P120 | P120 agagagacatatagagaaagagag |
|  |  | Sag29 P121 | P121 aacataagaataacagtaaagacaag |
| AT5G59310 | chr5:23942797-23942996 | LTP4 P136 | P136 gctttcgctttgaggttcttc |
|  |  | LTP4 P137 | P137 ggtcgggtgtggtttgag |
| AT1G72610 | chr1:27343387-27343528 | GLP1 Forward | TTC TGT GTC GCA AAC CTG AAA CGC |
|  |  | GLP1 Reverse | GAC AGC GGC GTT GAT GAT GTT TGT |
