# Supplementary figures and images for: Dynamic changes in genome-wide histone H3 lysine 4 methylation patterns in response to dehydration stress in Arabidopsis thaliana
Source: BMC Plant Biol. 2010 Nov 5;10:238. doi: 10.1186/1471-2229-10-238 (PMC3095321; doi:10.1186/1471-2229-10-238)

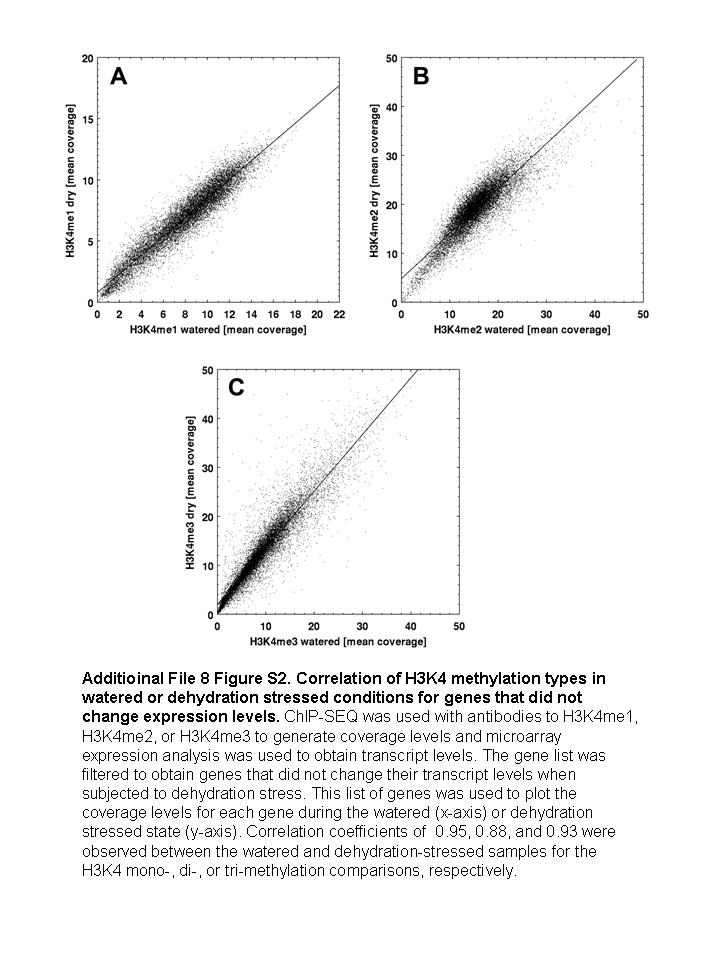

Supplement: Additional File 8 — Figure S2. Analysis of changes in H3K4 methylation types for genes that did not change expression when stressed. The levels of H3K4 mono-, di-, or tri-methylation for genes that did not show changes in transcript levels were plotted for the watered vs dehydration stress condition. [file 1471-2229-10-238-S8.TIFF]

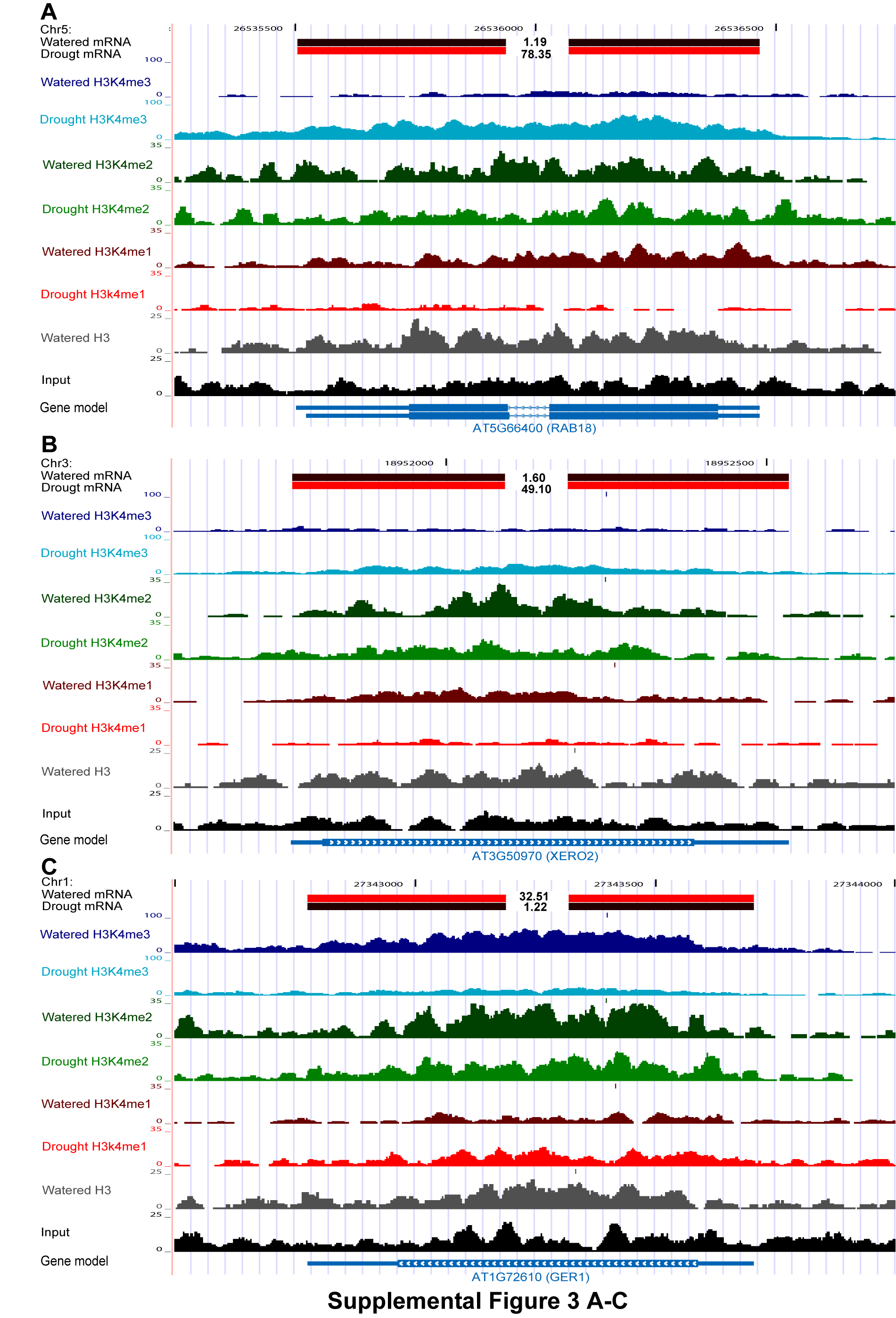

Supplement: Additional File 9 — Figure S3 A-C. Genome browser view of the chromosomal region genes with up- or down-regulated expression levels. The genome browser view of H3K4 mono-, di-, or tri-methylation levels for genes with increased or decreased gene expression are shown. [file 1471-2229-10-238-S9.TIFF]

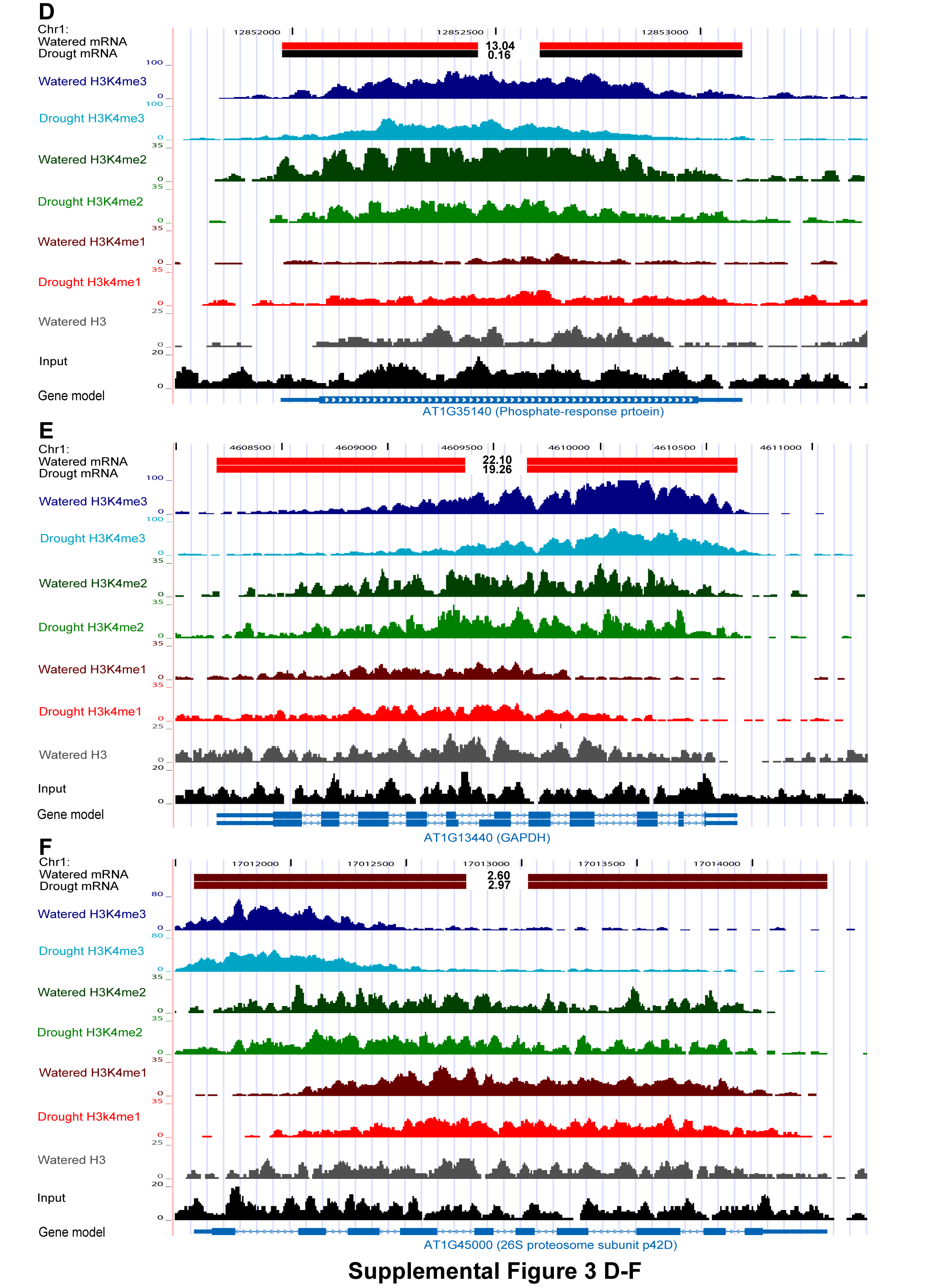

Supplement: Additional File 10 — Figure S3 D-F. Genome browser view of the chromosomal region genes with down- regulated or unchanged expression levels. The genome browser view of H3K4 mono-, di-, or tri-methylation levels for genes with decreased or no change in gene expression are shown. [file 1471-2229-10-238-S10.TIFF]
